# Macro and Microfluidic Flows for Skeletal Regenerative Medicine

**DOI:** 10.3390/cells1041225

**Published:** 2012-12-11

**Authors:** Brandon D. Riehl, Jung Yul Lim

**Affiliations:** Department of Mechanical and Materials Engineering, University of Nebraska-Lincoln, Lincoln 68588, NE, USA; E-Mail: brandon.riehl@huskers.unl.edu

**Keywords:** fluid flow, macroflow, 2D and 3D, microfluidics, bone, mechanotransduction, regenerative medicine

## Abstract

Fluid flow has a great potential as a cell stimulatory tool for skeletal regenerative medicine, because fluid flow-induced bone cell mechanotransduction *in vivo* plays a critical role in maintaining healthy bone homeostasis. Applications of fluid flow for skeletal regenerative medicine are reviewed at macro and microscale. Macroflow in two dimensions (2D), in which flow velocity varies along the normal direction to the flow, has explored molecular mechanisms of bone forming cell mechanotransduction responsible for flow-regulated differentiation, mineralized matrix deposition, and stem cell osteogenesis. Though 2D flow set-ups are useful for mechanistic studies due to easiness in *in situ* and post-flow assays, engineering skeletal tissue constructs should involve three dimensional (3D) flows, e.g., flow through porous scaffolds. Skeletal tissue engineering using 3D flows has produced promising outcomes, but 3D flow conditions (e.g., shear stress *vs.* chemotransport) and scaffold characteristics should further be tailored. Ideally, data gained from 2D flows may be utilized to engineer improved 3D bone tissue constructs. Recent microfluidics approaches suggest a strong potential to mimic *in vivo* microscale interstitial flows in bone. Though there have been few microfluidics studies on bone cells, it was demonstrated that microfluidic platform can be used to conduct high throughput screening of bone cell mechanotransduction behavior under biomimicking flow conditions.

## 1. Introduction

Skeletal regenerative medicine is of significant interest with a potential to improve the quality of life for those suffering from a variety of impaired skeletal conditions. Engineered bone tissue is in high demand considering a growing number of bone graft operations per year and a lack of low-risk donor materials. Further, treatments of other bone diseases (osteoporosis, Paget’s disease, osteogenesis imperfecta, osteomalacia, *etc.*) are also being sought with tissue engineering, since limited therapeutic molecular targets for these diseases are identified. Complicating the progress in the tissue engineering of bone is the wide range of mechanical properties, stages of development, and geometries of bone that should be replicated. There is not a uniform tissue we may label “bone” but rather a continuum of tissue mechanical properties that vary from patient to patient and even vary within a single bone. In the development and fracture repair processes, bone transitions from a woven or spongy structure to a highly organized, mineralized lamellar structure. Replicating the entire cascades of complex bone formation processes is still challenging.

One potentially very useful tool in bone tissue engineering is the mechanical stimulation from fluid flows. Throughout the bone are located lacunae and canalicular spaces in which interstitial fluids are filled and osteocyte network resides. Mechanical load produces interstitial fluid flows through the lacunae-canalicular channels and bone cells embedded inside the channels, such as osteocytes, sense, respond to, and communicate among cells the flow-induced mechanical signals. Mechanotransduction, the conversion of mechanical signals into biochemical cytosolic activities, under fluid flow stimulation has been proposed as a key element of bone homeostasis. For example, fluid flow has been shown to regulate bone cell proliferation and differentiation, release of bone stimulatory hormones, bone-like extracellular matrix (ECM) and mineral deposition, bone remodeling, and the quantity and quality of bone formed [1].

Fluid flow stimulation of cells has been conducted using various experimental set-ups. Studies used parallel plate devices, rocker plates, spinner flasks, rotating wall bioreactors, direct perfusion, *etc.* Fluid flow induces shear stress to the cells adhered to culture substrates and scaffolds. Depending on the geometry of the flow system, the stress profile can be determined by elementary fluid mechanics formulation for the case of relatively simple two dimensional (2D) flows, or via numerical method for the case of more complicated three dimensional (3D) flows. Note that we denote 2D flow as to have flow velocity varying only along the direction normal to the flow direction. For example, when cells are cultured on a glass slide and exposed to fluid flow within a flow chamber (e.g., flow between two parallel plates), a simple fluid mechanics formula predicts a parabolic velocity profile along the height of the chamber when we project the flow from the front face of the chamber. This profile can be called 2D flow, since the profile theoretically does not vary along the 3rd axis, *i.e.*, along the depth of the chamber. In contrast, any complex flows following 3D geometries, e.g., flow through porous scaffolds, are denoted as 3D flows in this review. Studies involving macroflows will be reviewed depending on the dimensionality (2D and 3D). The 2D flow studies have been dedicated to reveal the molecular mechanism of cell sensing of and response to flow, while 3D flow studies more to provide improved engineered bone constructs. Further, recent microfluidics approaches will be highlighted, although few studies on microfluidics directly relevant to bone tissue engineering exist so far. For a more in-depth review on general microfluidics and cell behavior, refer to references [2,3].

## 2. Macroflows for Bone Cells

### 2.1. Two Dimensional Macroflows

The 2D fluid flow studies have demonstrated that bone cells are highly sensitive to fluid flow-induced shear stress stimulation and that mechanosensitive bone cell responses are dependent on flow regimen (shear stress magnitude, oscillatory or steady, flow time, resting period), cell culture substrate, and environmental cues from soluble factor and co-cultured cells [1]. Flow regulation of cell growth, differentiation and gene regulation, bone matrix deposition, and cellular communication has been demonstrated using 2D flow studies, while there is relatively little consensus over the loading and scaffold combinations useful for engineering 3D bone tissue constructs [4,5]. Though the comparison of reported 2D data may also not be completely feasible due to different methods of applying the flow and variations in culture conditions, 2D assay remains as a powerful tool for revealing the mechanism of flow control of bone cells due to easiness of *in situ* and post molecular biology assays. For example, *in situ* measurement of flow shear-induced cytosolic calcium, Ca^2+^, evolution can be conducted during the flow using 2D cell culture between parallel plates and fluorescent imaging on inverted microscope. In this section, we will highlight important aspects of bone forming cell responses to fluid flow revealed through 2D flow assays.

Osteocytes (embedded and interconnected bone cells), osteoblasts (bone forming lining cells), osteoclasts (bone resorbing cells), and their progenitor cells serve unique roles in bone remodeling and homeostasis. Consequently, these cells may respond differently to fluid flow. For example, osteocytes and osteoblasts displayed differential responses to oscillatory and steady flows with varying stimulus time, shear stress, and frequency [6,7]. Specifically, osteocytic network responded in cytosolic Ca^2+^ signaling to fluid flow regardless of the magnitude of shear stress, whereas the response of osteoblastic network significantly depended on the strength of the flow [6]. But still, osteocytes and osteoblasts share many aspects of structural and molecular responses when exposed to flow. Osteocytes under flow showed cytoskeletal remodeling with stress fiber realignment and increases in ATP release, Ca^2+^ signaling, alkaline phosphatase (AP) activity, Cyclooxygenase-2 (Cox-2), and osteopontin (OP) gene expression [4,6,7,8]. Similarly, osteoblast response to flow often resulted in increased proliferation and osteogenic gene expression and changes in cytoskeletal organization and stiffness with upregulation in signaling molecules mentioned for osteocytes [7,9,10].

Osteocytes form interconnected networks throughout the bone, sensing mechanical force in lacunae-canalicular channels and directing bone remodeling. The osteocytic signaling activity has been proposed to operate through different modes depending on the target signaling cell type. For example, osteocytic cell processes are connected to each other via gap junctions, which processes are further connected to bone forming osteoblasts. The cell-to-cell communication from osteocytes to osteoblasts was proposed as one of the mechanisms of new bone formation [11]. On the other hand, mesenchymal stem cells (MSCs) in the bone marrow tend to respond through paracrine signaling when osteocytes are stimulated [12]. Among these, the primary response of osteocytes to fluid flow, congruent with their role as a primary bone mechanosensor, involves cell-cell interaction-mediated modulation of the other bone cells. This has been shown with fluid flow studies using co-culture and conditioned media. Osteocytes, when stimulated by fluid flow, could regulate the activity of osteoblasts through gap junction intercellular communication (GJIC) [13]. This was shown using the flow between two parallel disks, in which osteoblasts connected to flow-stimulated MLO-Y4 osteocytes via gap junctions displayed significant upregulation of AP activity, whereas this response was lacking in osteoblasts merely co-cultured with non-flowed osteocytes or cultured in conditioned media from flowed-osteocytes.

Osteocytes subjected to fluid flow have also been shown to downregulate the activity of bone resorbing osteoclasts and stem cell commitment to osteoclast. For example, bone marrow stromal cells co-cultured with osteocytes could form osteoclasts in static culture, but their osteoclastogenesis was inhibited if osteocytes were stimulated by fluid flow [14]. This was achieved through elevated matrix extracellular phosphoglycoprotein, which in turn upregulated the expression of osteoprotegerin (OPG), an osteoclast inhibitor. Similarly, when ST-2 bone marrow stromal cells were co-cultured with RAW 264.7 monocytes, they formed osteoclasts under static culture, as assessed by tartrate-resistant acid phosphatase (TRAP) assay [15]. However, TRAP was significantly downregulated under oscillatory fluid flow, accompanying the downregulation of receptor activator of NF-κB ligand (RANKL) and upregulation of OPG. Together, it may be concluded that osteocytes play a role as a forefront mechanosensor under flow stimulation, enhancing bone formation by stimulating osteoblast and inhibiting bone resorption by downregulating osteoclastogenesis.

**Figure 1:**
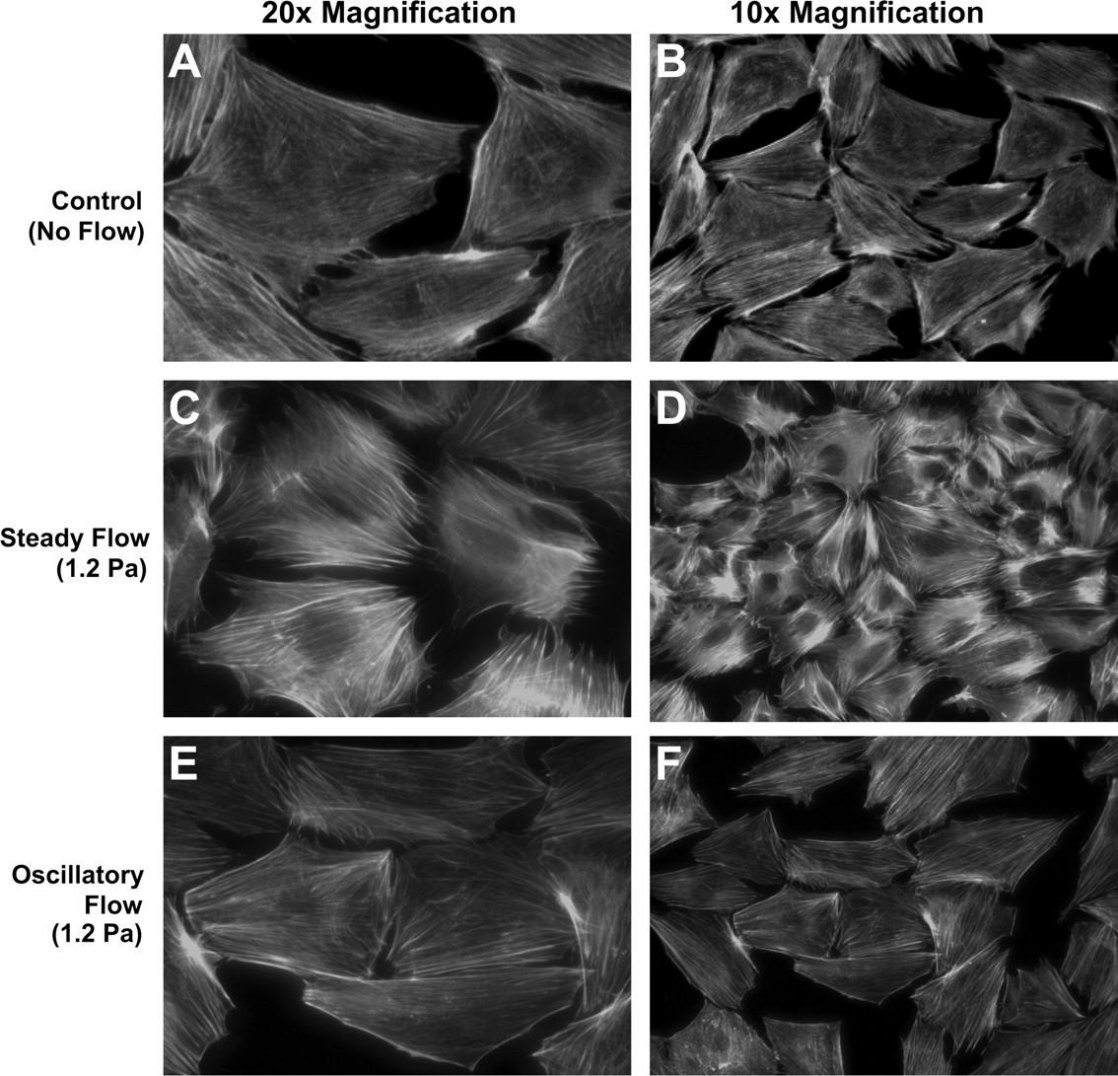
MC3T3-E1 cell response to steady or oscillatory fluid flow in 2D flow apparatus. (**A**,**B**) Unorganized F-actin stress fibers in static culture. (**C**,**D**) Organized stress fibers in response to 1.2 Pa steady flow. (E,F) No distinct stress fiber formation under 1.2 Pa oscillatory flow at 1 Hz.Reprinted with permission from the American Physiological Society (Malone * et al.* [16]).

Osteoblasts are guided by osteocytes, as described above, but it is notable that osteoblasts have also been shown to be directly responsive to fluid flow cues. Typical osteoblast responses to fluid flow involve proliferation, differentiation, cytoskeletal remodeling, and regulation of factors important for bone formation including AP, osteocalcin, and prostaglandin E2 (PGE2) as well as gene regulation involved in bone formation [9,10,16,17,18,19,20]. Osteoblast responses varied with peak shear stress, frequency, time, and culture substrate. Malone *et al.* [16] observed that steady or oscillatory flow differentially regulated stress fiber formation, e.g., F-actin stress filaments were significantly organized in response to continuous flow but not by oscillatory flow, both at 1.2 Pa stress (Figure 1). Fluid flow caused stress fiber remodeling by reorganizing F-actin fibers and potentially increased the abundance of bound proteins, e.g., α-actinin, filamin, and vimentin.

How to direct and guide stem cell lineage commitment and differentiation toward osteoblastic lineage has been the critical topic in stem cell-based bone tissue engineering. The 2D flow assays have provided templates for studying mechanical direction of stem cell osteogenesis. Many studies using 2D flows have demonstrated that fluid flow stimulation of MSCs increased AP release and upregulated key osteogenic genes/transcription factors such as Runx2, bone morphogenetic protein-2 (BMP-2), OP, and bone sialoprotein [21,22,23]. These changes were often dependent on shear stress level and pre- and post-culture time [24]. In addition to mechanical induction of MSC osteogenesis, MSCs also showed enhanced osteogenic commitment through soluble cues from osteocytic culture, as pointed out above [12]. MSCs cultured in media from fluid flow-stimulated osteocytes upregulated Runx2, OP, and Cox-2 gene expressions by up to 2 fold relative to MSCs cultured in media from non-flowed osteocytes. Interestingly, MSCs cultured with media from flowed osteoblasts did not induce osteogenesis, highlighting the unique role of osteocyte signaling in guiding MSC lineage commitment to bone cell phenotype.

Since stem cells *in vivo* are subject to numerous cues other than mechanical signals, including soluble factors and niche microenvironments, the co-use of the other factors with flow may provide synergistic environments for optimal induction of MSC osteogenesis. Further, since tissue engineering uses scaffolds for cell adhesion and growth, the effects from cell culture substrate will also play a role. We recently showed that nanoscale bone-mimicking substrate topographies may be beneficial for inducing MSC osteogenesis [25]. MSC osteogenic fate would also be determined via the use of osteogenic induction cocktail and patterned cell-adhesive ligands [26]. In relation to fluid flow, we demonstrated that fluid flow-induced cell signaling in stem cells may be affected by cell-substrate interaction [27]. On specific scale (10-20 nm high) nanoisland textures, human mesenchymal stem cells (hMSCs) displayed increased mechanosensitivity to 2D flow, e.g., greater percentage of cells showing Ca^2+^ upregulation under fluid flow and greater Ca^2+^ jump for responding cells, relative to cells on the flat control. Since cytosolic Ca^2+^ signaling is one of the primary molecular events for bone forming cells under flow [1], our data suggest that flow-induced MSC fate direction may be modulated via manipulating cell-substrate interaction. Similar effect was also shown with osteoblasts. Takai *et al.* [28] showed that bone cell response to flow was dependent on the presence of ECM protein, e.g., flow-induced PGE2 secretion in MC3T3-E1 cells was greater when cultured on fibronectin relative to glass. Substrate microarchitecture also modulated osteoblast response under fluid flow. Osteogenic factors including AP, osteocalcin, transforming growth factor (TGF)-β1, and PGE2 were upregulated in MG63 cells under fluid flow, but this was observed only on microscale roughness surfaces but not on smooth surfaces [29].

The effects of cell-substrate interaction on bone forming cell mechanotransduction suggest the mediatory role of focal adhesion and related signaling. Cells form focal adhesion via ECM ligand-integrin receptor binding with many proteins and kinases, including vinculin, paxillin, talin, α-actinin, focal adhesion kinase (FAK), *etc.*, associated with integrins at focal adhesion sites [30]. When cells are stimulated by fluid flow-induced shear stress, focal adhesions (cell anchoring sites) will behave as the first resistant sites. Thus, relative abundance of focal adhesion and the strength of related signaling activities may determine cellular responsiveness to flow stimulation. We showed that integrin-FAK profile is a sensitive marker for revealing cell-substrate interaction [31,32]. Specifically, human fetal osteoblastic (hFOB) cells displayed upregulation in integrin (αv but not α5, β1, β3) expression and FAK phosphorylation at pY397 when cultured on 10-20 nm deep nanopit textures [32]. This may be positively correlated with stem cell mechanotransduction data as described above, though the cell type is different. Upregulated focal adhesion activities on specific nanotextures may be responsible for the promoted Ca^2+^ response under flow stimulation. Recent studies proposed the potential role of FAK as a dynamic mechanosensor under flow. Osteoblasts with disrupted FAK exhibited impaired mechanical responses under 2D fluid flow in several mechanosensory markers including extracellular signal-regulated kinase (ERK) [33,34].

Cytoskeletons, which are anchored at focal adhesions, and related tension signaling may also play a vital role in fluid flow-induced cell mechanotransduction. It was observed that disrupting the cytoskeletons in bone cells by addition of nocodazole and cytochalasin D inhibited fluid flow-induced production of mRNAs involved in ECM remodeling, Col-1, matrix metalloproteinase (MMP)-1, and MMP-3 [35]. While shear-induced changes in cytoskeletal composition, organization, and stiffness have been relatively well established [10,16], the role of tension signaling such as RhoA/RhoA kinase (ROCK) in bone forming cell response to fluid flow was only recently highlighted. C3H10T1/2 MSCs displayed fluid flow-induced upregulation in Runx2, Sox9, and PPARγ mRNA, suggesting a potential of fluid flow in inducing MSC fate to multiple lineages including osteogenesis, chondrogenesis, and adipogenesis [23]. If focused on osteogenesis, RhoA/ROCK activation and flow additively upregulated Runx2, suggesting that cell tension signaling may work as a vital mechanosensor in the flow induction of MSC osteogenesis.

Additionally, macroflow assays in 2D have been very useful in revealing the characteristics of healthy and diseased bone cells. Marked differences in response to fluid flow have been observed in osteoporotic and osteoarthritic cells *vs.* healthy cells. While osteoporotic cells displayed similar initial responses to flow in PGE2, nitric oxide (NO), and TGF-β relative to healthy cells, they lacked 24 h-lasting regulation present in healthy cells [36]. Differences were also observed between osteoporotic and osteoarthritic cells [37]. Flow-induced NO expression was greater in osteoporotic cells relative to osteoarthritic cells, whereas PGE2 expression was relatively greater in osteoarthritic cells. Further, in osteoporotic cells NO increased rapidly and saturated at medium shear stress (0.6 Pa at 5 Hz), whereas in osteoarthritic cells NO increased steadily and was the highest at high shear stress (1.2 Pa at 9 Hz). It was proposed that differences in bone defects for osteoporosis and osteoarthritis may lead to altered mechanical cell responsiveness to flow stimulation.

Through mechanotransduction studies using 2D fluid flow assays, in which *in situ* and post molecular biology assays are feasible, it is hoped that one may reveal the cellular and molecular mechanisms of bone and stem cell sensing of and response to fluid flows and also identify mechanisms of bone degeneration to provide insight for novel molecular therapeutic targets for bone diseases.

### 2.2. Three Dimensional Macroflows

Moving to 3D is an inevitable step in engineering functional bone tissue replacements. The 2D flow studies may reveal the molecular mechanism of bone cell mechanotransduction under flow and obtained data may be useful for guiding 3D studies. However, a potential dilemma exists that the data obtained from 2D flow assays may not be directly translated into 3D situations. For example, studies demonstrated that cells respond to shear and tensile forces differently under 2D *vs.* 3D conditions [38,39,40,41]. Specifically, cells may require less shear stimulation in 3D to achieve similar response as in 2D [38]. Therefore, the application of 2D data to 3D flows for tissue engineering purpose should count 3D situations, e.g., variable shear stress conditions, altered transport of soluble molecules, and modified cell-substrate and cell-cell interactions. This section will highlight important aspects of bone forming cell responses to 3D flows.

Similar to 2D studies, 3D flow stimulations of bone forming cells exhibited fluid flow-induced osteogenic differentiation via upregulation of important osteogenic genes. This resulted in enhanced deposition of bone-relevant ECM and minerals within 3D engineered tissues. Studies showed that flow through 3D geometries may assist cell function via modulated fluid mechanics. Specifically, perfusion bioreactors could aid in stimulating cells through applying shear forces and also via providing an effective way for nutrient, waste, and signaling molecular transport. It is thus generally accepted that 3D perfusion flow has a great potential for tissue engineering, but not all perfusion-scaffold combinations led to desired outcomes in osteogenesis. For instance, for some scaffolds static culture showed even greater osteogenic induction than perfusion culture [42]. Other parameters of 3D fluid flow including flow rate, cyclicity, frequency, scaffold porosity, and media composition also affected bone forming cell responses [43,44,45,46].

One very important aspect of 3D flow is the correlation between shear stress and transport phenomena. It was demonstrated that shear stress applied to the cells and chemotransport were not only dependent on the flow rate but also determined by scaffold porosity and the method of applying flow [47]. Considering that solute transport is driven by pressure difference in porous bone due to mechanical loading, studies using tracers attempted to quantify transport phenomena under flow. It was demonstrated solute transport was enhanced by up to 100 fold under oscillating fluid flow [48,49,50]. Flow through 3D porous scaffold is very complex, which requires computational fluid dynamics for estimating the shear stress profile and chemotransport dynamics [51]. One starting point in assessing complex 3D flow includes the assessment of flow direction relative to the scaffold. It was observed that parallel flow configuration (around the scaffold) preserved hMSC progenicity and proliferation potential with retained ECM proteins, while transverse flow configuration (through the scaffold) induced osteogenic differentiation as was visible in bone marker expression and calcium deposition [52]. However, it is not clear whether this was due to altered shear stress profile or from difference in chemotransport in the scaffold.

Distinguishing cell response to shear stress and chemotransport is difficult as the method of shear stress stimulation and the mechanism of chemotransport are coupled. Studies have attempted to decouple this relationship by adding dextran to the flow media to change the viscosity. For example, with the addition of dextran the same flow rate produces higher shear stress due to increased viscosity. So the effect of varying shear stress can be tested under the same rate of mass transport (flow rate). Alternatively, shear stress can be maintained the same even with changing the flow rate by differentially adding dextran. So the effect of varying mass transport can be examined at constant shear stress level. Li *et al.* [53] used dextran to study each effect from shear stress and mass transport in 3D flow. They observed increasing flow shear stress accelerated MSC osteogenic differentiation and improved mineralization. However, interestingly, increasing mass transport inhibited the formation of mineralized ECM. Such a test using dextran was also attempted for 2D fluid flow. For instance, Riddle *et al.* [54] proposed that chemotransport may be a prerequisite to shear-induced mechanotransduction in stem cells. Bone marrow stromal cells exhibited greater Ca^2+^ and ATP releases with increasing rate of chemotransport (flow rate) under the same shear stress. Starving the cells by substituting Hanks’ buffered salt solution for standard media significantly decreased cell response to flow, which effect was not changed even when the flow rate was increased. Taken together, while 2D and 3D studies suggested some individual roles of shear stress and transport, data are not consistent and the correlation between them is not fully understood yet.

In addition to differences in flow parameters, there are more differences in 2D *vs.* 3D. Simply changing milieus from 2D to 3D, even under static culture, may have significant effects in a variety of cell behavior, including integrin-mediated focal adhesion, actin skeletal structure and cell tension, cell growth, and differentiation [41,55]. Importantly, cell-cell network formation is hugely different for 2D *vs.* 3D culture. The potential difference in cell-cell communication may in turn affect bone forming cell response to fluid flow, since mechanical signals sensed by one cell will be transmitted differently to the adjacent cells. Though the differential response has not been fully revealed, one may speculate that 3D flow within a scaffold mimicking actual bone tissue may provide potentially more biomimetic stimulatory effects on bone formation relative to 2D flow.

The use of stem cells and fluid flow-based 3D bioreactors has been the prominent strategy for skeletal tissue engineering. MSCs cultured in 3D dynamic spinner flask culture showed significant upregulations of early osteogenic commitment markers (Runx2, BMP-2, COL1A1) and osteogenic differentiation markers (AP activity, mineralization) compared with static culture [56]. The guidance of stem cells toward osteogenic differentiation in 3D bioreactors has been shown to depend on the flow regimen. Liu *et al.* [45] observed that intermittent flow (stress alternating from 4.2 dynes/cm^2^ for 1 h to 0.34 dynes/cm^2^ for 11 h) for 14 days significantly enhanced osteogenic gene expression along with increased ERK1/2 and FAK phosphorylation relative to cells cultured in a continuous flow of 4.2 dynes/cm^2^or static control. The other study demonstrated that higher flow rates stimulated hMSC differentiation in AP activity and calcium deposition, while low flow rate supported proliferation and fibronectin secretion [38].

Progresses have been made in generating biomimetic bone grafts *in vitro* through the use of demineralized bone/synthetic scaffolds seeded with bone marrow-derived MSCs and adipose-derived stem cells. Further, the adoption of 3D perfusion bioreactors made it possible to overcome the mass transport limitation of static culture in which cell viability is limited to areas close to the scaffold surface. Perfusion flow also created high quality bone grafts by allowing uniform and enhanced ECM deposition and mineralization with increasing perfusion rate [57,58,59]. Fröhlich *et al.* [57] cultured human adipose-derived stem cells in decellularized bone, with and without osteogenic induction media, in a perfusion bioreactor for up to 5 weeks. Stem cells under flow still required osteogenic medium for complete differentiation. However, cell distribution and collagen deposition in 3D flow system were uniform, allowing cells to create a matrix environment mimicking native bone by depositing minerals inside the scaffold.

**Figure 2:**
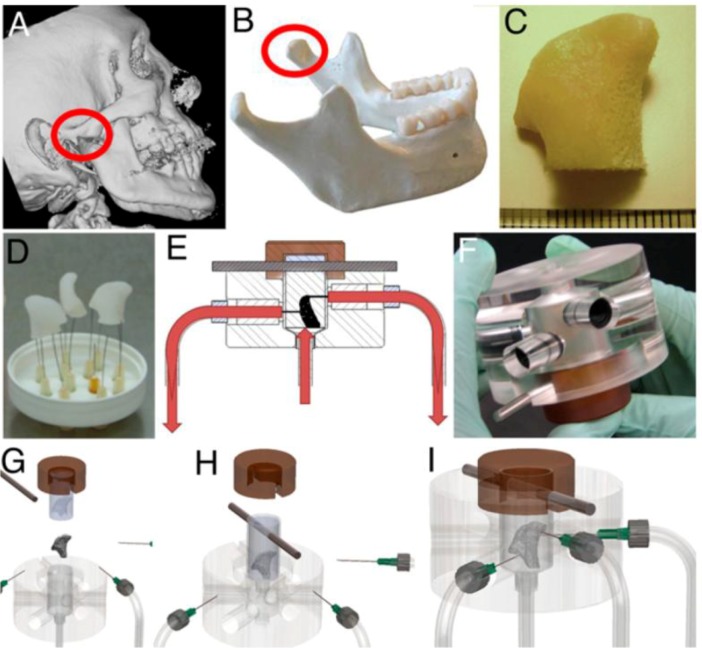
Tissue engineering of anatomically shaped bone grafts using 3D flow. (**A**,**B**) computed tomography (CT) images were used for the reconstruction of exact geometry of temporomandibular joint (TMJ)condyles. (**C**) Scanned data were used tomachine TMJ-shaped scaffolds from fully decellularized trabecular bone. (**D**) Image of produced scaffolds. (**E**) Human mesenchymal stem cells (hMSCs) were statically cultured for 1 week in the scaffolds, and then theperfusion was applied for additional 4 weeks.(**F**) Image of the perfusionbioreactor. (**G**–**I**) Keysteps in bioreactor assembly. Reprinted with permission from the National Academy of Sciences of the United States (Grayson * et al.* [60]).

Grayson *et al.* [60] made significant advances in patient-specific bone tissue engineering by using a 3D flow bioreactor having a chamber in the exact shape of a human temporomandibular joint (TMJ). They proposed to precisely match the geometry of the target bone tissue to be engineered from the computed tomography (CT) scan of the patient (Figure 2). The scanned data were incorporated into MasterCAM software to machine TMJ-shaped scaffolds from fully decellularized trabecular bone. When hMSCs in the scaffold were exposed to flow, a polydimethylsiloxane mold was placed around the scaffold to ensure the flow perfusion through the scaffold instead of flow around the scaffold. Perfusion under this condition significantly increased cell proliferation and mineralized matrix production, e.g., lamellar-like bone with new osteoid formed, relative to static culture. This approach may become even more powerful if combined with recent developments in 3D bioprinting technique that can be used to fabricate scaffolds with desired 3D geometry and porosity from user-defined materials [61].

The use of decellularized bone scaffold, regardless of its promising data, may not be ideal. Autograft is a good candidate but has certain drawbacks of limited supply, donor site morbidity, pain, and prolonged rehabilitation. In case of allograft, transmittance of donor pathogens and triggering of host immune response may be problematic. Thus, the development of advanced artificial scaffolding materials is critically needed for widespread clinical use. Although material constraints may vary from application to application, the goal of engineering a new bone tissue requires that materials are selected to closely mimic native bone tissue in the long run. Polymers, ceramics, and metals have been tested as osteoinductive bone scaffold materials with varying degrees of success and acceptance. Metals, though proven very useful for orthopaedic bone fixatives, do not meet the requirement of tissue engineering since they cannot be degraded/remodeled by bone cells as degradable polymers and hydroxyapatite would be [62]. Also, bone scaffolds should have sufficient initial mechanical strength to prevent stress shielding effect, and should support cell attachment and angiogenesis for improved bone physiology while regeneration [63]. In relation with fluid flow stimulation, it may be beneficial if the scaffold increases the mechanosensitivity of the cells, as suggested from 2D studies [27,28,29]. Considering our data showing increased Ca^2+^ signaling under 2D flow for hMSCs seeded on specific nanotextures [27], modifying surface texture of scaffolds to have nanotopography may increase the mechanosensitivity of cultured bone forming cells to 3D flow stimulation. Increased cell mechanosensitivity may finally contribute to enhanced osteogenic differentiation [25]. Sensitizing cells to be more responsive to flow cues may be also beneficial for the case where only limited shear stress range is available. For instance, for the flow through 3D scaffold with less porosity and small pore size where high volume flow rate is difficult to achieve, cellular mechanical sensitization may allow even lower shear stresses to function as a potent stimulator of bone forming cells [27].

## 3. Microfluidics for Bone Cells

Microfluidics is a relatively new field of study that has a potential to provide tremendous opportunities for investigating skeletal regenerative medicine and bone cell physiology as well. The biggest advantage of the microfluidics, in comparison with macroflows, is microscale flow channels, having sizes comparable to those of *in vivo* interstitial flows, can be fabricated through which laminar flow can be flowed in a controlled manner. Note, flow in the microfluidic channel tends to be laminar, but not turbulent, due to low Reynolds number given by the small channel size. Since the dimensions of microchannels mimicking *in vivo* length scales are small, less reagents and cells per test are needed. Further, microfluidics can be designed for high throughput screening with improved automation, which greatly increases the speed and efficiency of measurement and evaluation. Even the device capable of capturing and operating on down to single cell level could be fabricated [64,65], which is infeasible for macroflows.

**Figure 3:**
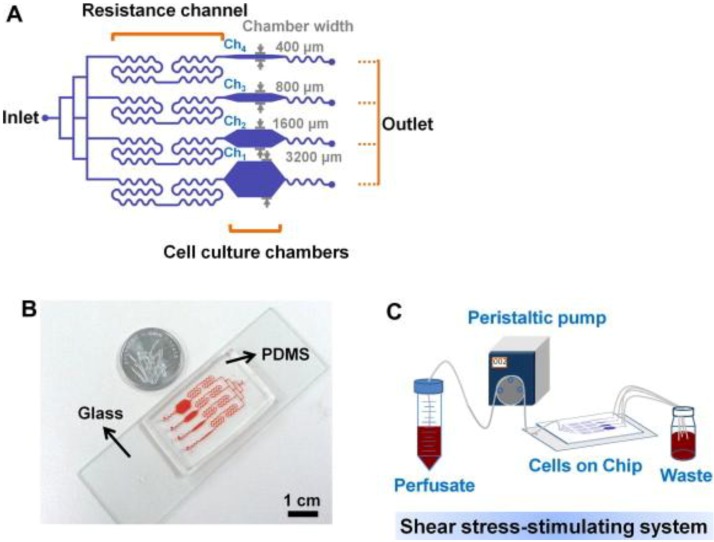
Multishear microfluidic device used for assessing bone cell response to fluid flow at varying shear stresses. (**A**) Flow path in microfluidic chip with resistance channels. The channel with narrow chamber width (e.g., 400 μm) produced higher shear stress. (**B**) Size comparison of chip with a coin. (**C**) System overview of media flow path. Reprinted with permission from Elsevier (Kou *et al.* [66]).

For bone cells, not many studies on microfluidics have been reported. Recently, Kou * et al.* [66] designed a multishear microfluidics template for assessing bone cell response to fluid flow, serving as a counterpart for conventional macroflow assays. They designed multiple microfluidic channels via which multiple shear stresses can be applied to the cultured osteoblasts in a simultaneous manner (Figure 3). Due to the size and clarity of the microfluidic chip, the device was mounted on a microscope and cytosolic Ca^2+^ release under varying shear stresses could be imaged simultaneously. Although obtained Ca^2+^ response data showed the same trend with those from macroflows, e.g., greater cytosolic Ca^2+^ response with increasing shear stress, this study opened a new opportunity to assess bone cell response to fluid flow at varying shear stresses. Other studies showed that osteogenesis could be induced by microflow and incorporated soluble factors. Leclerc *et al.* [67] studied osteoblast response to 0, 5, and 35 μL/min flow in a 3D microchannel and showed that AP activity could be enhanced by 7.5 fold at 5 μL/min compared with static control. Jang *et al.* [68] designed a drug screening device and observed that microfluidic flow (0.07 dyne/cm^2^ at 0.2 μL/min) and BMP-2 cue flowed through the channel could induce osteogenic differentiation of MC3T3-E1 cells.

An interesting attempt was made to utilize microfluidic device as a magnetic bead impact generator to apply the other type of physical stimulation to the cells. Osteoblasts in microfluidic channels were bombarded with magnetic beads controlled by microelectromagnetic fields to test if bombardment had differential effect depending on the cell phase [69]. The growth rate of MC3T3-E1 cells increased significantly by up to 193% with bombardment of 4.5 μm beads at 1 MHz (resulting in a 0.06 N force) when cells were in G1 phase, but it was not significantly affected for cells in S or G2 phase.

**Figure 4:**
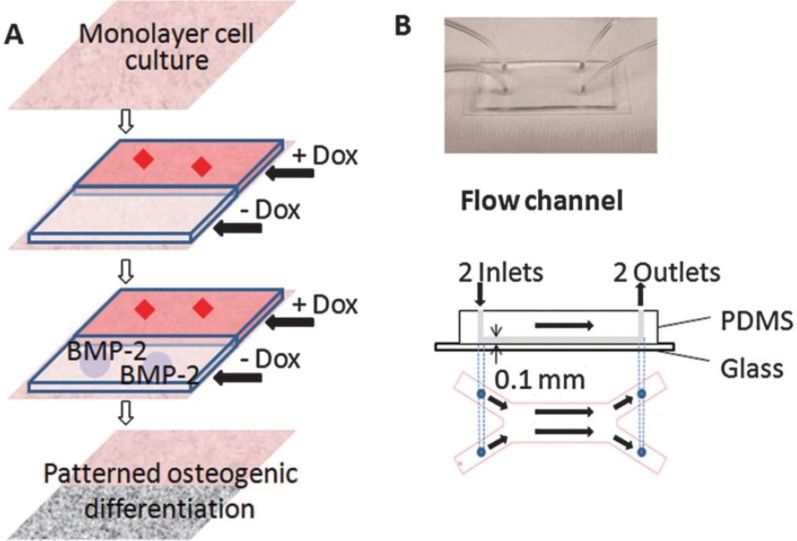
Patterning osteogeniccell differentiation using two layers of microflows. (**A**) Cultured cells were exposed to laminar flow with two streams, with and withoutDox, a bone morphogenetic protein-2 (BMP-2) inhibitor. The patternedDox layers led to patterned BMP-2 expression, thereby leading to patterned osteogenic differentiation. (**B**) Image and schematic of the flow channel. Reprinted with permission from the Royal Society of Chemistry (Zhang * et al.* [71]).

Other studies utilized gradient microfluidic channels to test cell adhesion and differentiation. These studies did not primarily focus on applying mechanical stress to the cells but used microfluidics to produce gradient channels. The laminar nature of microfluidics can be taken advantage of to create gradients in parallel flows formed within a single chamber. In microfluidic pH gradient channels having polyelectrolyte multilayer substrate, MG63 osteoblast adhesion was greater on basic pH regions relative to acidic regions [70]. In the study by Zhang *et al.* [71], a microfluidic device having two distinct laminar layers was created within a single channel and Doxycycline (Dox), a BMP-2 inhibitor, was introduced into one of the layer (Figure 4). C3H10T1/2 murine MSCs in the Dox-introduced layer showed undifferentiated cell phenotype, while cells in the other layer showed flow-induced osteogenic markers and calcium deposition. Using this fluidic platform, osteoblastic differentiation profile can be spatially patterned and the effects of new pharmacological factors on bone cell differentiation may be screened.

Microfluidic devices recently found new applications as high efficiency/accuracy detection tools. A device having 3D micropillar electrode and PDMS micropillars in serpentine microchannels was created for improving enzyme-linked immunosorbent assay (ELISA) [72]. Using this device, the efficiency of detecting bone cell differentiation marker was significantly increased. In detecting and analyzing cells, capturing of single cell within microfluidics is now capable, e.g., a dielectrophoretic ring trap could capture a fluorescent-expressing osteoblast [65]. Chen *et al.* [73] took this a step further by forcing cells through a constricting channel and measuring the electrical impedance and time to travel. A neural network was trained to correctly distinguish between osteocytes and osteoblasts based on these properties with 94% success rate. This system may be useful for quantifying the ratios of cells from clinical samples or may be modified to ensure that cells are not damaged during the sorting process.

Microfluidic devices are also used to screen biomaterials relevant to osteoblast culture and infection. A high throughput screening template was developed to find substrate combinations for preventing bacterial infection while promoting osteoblastic differentiation and calcium deposition [74]. Among microchannels consisting of various biomaterial combinations, a channel consisting of patterned antibiotic biphasic calcium phosphate nanoparticles on poly(lactic-co-glycolic) acid matrix promoted osteoblastic cell growth and calcium deposition. The same group tested the effects of Staphylococcus epidermidis on osteoblast adhesion and viability on Ti alloy surfaces using a microfluidic co-culture environment [75]. They showed that over the time course the bacteria altered the microenvironment creating an acidic condition, which caused the loss of osteoblast viability. Another microfluidics application includes the assessment of cell-cell interaction via a fluidics-based co-culture system [76]. Macrophages and osteoblastic cells were grown in separate wells, upstream and downstream respectively, in a microfluidic device. An inflammatory response was then triggered by placing polymethyl methacrylate (PMMA) debris in the upstream media, which caused macrophages to release tumor necrosis factor (TNF)-α. Osteoblasts in the downstream wells responded to TNF-α by releasing bone remodeling molecule, PGE2. On the other hand, osteoblasts showed little response to direct PMMA stimulation in the well. Again, high throughput testing using multiple channels and conditions was possible.

## 4. Implications in Skeletal Regenerative Medicine

There have been many interesting studies on skeletal regenerative medicine adopting fluid flow. However, the flow regulation of bone forming cells and optimal flow-controlled tissue engineering conditions have not been fully revealed. Many 2D and 3D studies have demonstrated principles of flow control of bone cells and attempted to find potential mechanotransduction targets for therapeutic treatments of bone diseases. Although 3D flow is the primary tool for engineering bone tissue, 2D flow study is useful for understanding underlying molecular mechanisms. Recent microfluidic approaches may unveil unprecedented data on such mechanism by providing templates for high throughput and high resolution screening of bone cells under flow.

The use of relatively simple fluid mechanics as in 2D flow chambers aids mechanistic studies. The simple 2D flow geometry is useful in determining shear stress regimes/criteria, e.g., shear stress level, steady *vs.* oscillatory, duration of flow, insertion of resting period, *etc.*, favorable for bone forming cell functioning without potential interference from complex 3D flows. However, signaling data revealed through 2D flow may not be applied the same in tissue engineering using 3D flows. The regulation of molecules involved in mechanosensing events (ATP, Ca^2+^, ERK, PGE2, *etc.*), factors involved in bone formation (Runx2, AP, BMP-2, OP, *etc.*), and osteoclast inhibitor (OPG) in bone forming cells revealed through 2D flow studies are all significant findings. Data also suggest that focal adhesion, cytoskeletal development, and related signaling cascades play mediatory roles in cells sensing and responding to fluid flow. Additionally, potential upregulation of cell mechanosensitivity on specific culture substrate, as observed in our and other group’s studies using 2D flows, may suggest a promising implication on how to achieve successful bone tissue engineering using 3D fluid flow stimulations.

Studies have achieved well-mineralized bone grafts using 3D flows but these grafts still lack structures when compared with native bone. Perfusion serves to supply the inner portions of the graft with mass transport *in vitro* but once implanted, the inner cells are often cut off from nutrient and waste exchange. Co-culture of osteogenic cells and vasculogenic cells in a 3D perfusion system may solve this problem, seeing that implanted constructs formed vasculature within the bone [77]. However, still, a tissue engineering strategy with 3D flow stimulation conditions to cover all graft cases including larger bone constructs is required. Also needed are studies that identify the effects of co-culturing osteocytes on osteoblasts and stem cells, since cell-cell communication, either via contact though gap junctions or through soluble signals, has been shown to play critical roles in bone homeostasis. Many scaffold materials and cell sources have been attempted and found successful under various cell seeding and flow conditions. Also needed in this case are more systematic studies under unified 3D flow conditions and profiles so that the data may be compared among studies. In addition, while the use of established cell line cells helps the comparison among studies, the data may not be relevant to tissue engineering using patient-obtained cells.

Fluid flow stimulation of cells using macroflows is now revisited using microfluidics at a more biomimetic flow condition and at a high throughput testing efficiency. Further, automated selection and sorting of cells in microfluidic chips may lead to patient-specific optimization of cell culture conditions for bone graft engineering. Devices have been attempted for AP activity and other differentiation markers to be monitored autonomously in high throughput chips, which would allow feedback about patient cell response to culture conditions. Microfluidic devices also provide ideal platforms for performing high throughput screening of mechanotransduction signaling under flow, studying dose-dependent bone cell response under gradient conditions, and conducting co-culture studies.

## 5. Fluid Flow Effects on Other Lineages

The discussion to this point has focused on fluid flow-driven differentiation and guidance of bone remodeling cells. It is important to note that fluid flow is an important regulator of other tissues and cell types throughout the body. For example, the magnitude of flow shear stress was found to modulate stem cell migration to wound sites, which may also have implications for preosteoblast recruitment *in vivo* [78]. Particularly relevant to this review is the stem cell fate decision to adipogenic and chondrogenic lineages which are other cells in the skeletal system. Adipocytes in the bone marrow have been suggested to respond to temperature and pressure differences [79], and MSCs *in vitro* have been guided to adipose lineages through cell confinement on small island patterns and downregulation of cytoskeletal tension [80]. Some studies indicate that adipogenic and chondrogenic differentiations are regulated by fluid flow. For instance, oscillatory fluid flow could induce the upregulation of not only Runx2 but also PPARγ and Sox9, indicating flow signal may have the potential to regulate transcription factors involved in multiple lineages, osteogenesis, adipogenesis, and chondrogenesis [23]. On the other hand, the other study showed fluid flow could reduce the expression of adipogenic marker, lipoprotein lipase, in bone marrow stromal cells [21]. Many studies showed evidence of flow-induced upregulation of chondrogenesis. MSCs cultured in 3D scaffolds with chondrogenic medium subjected to perfusion flow showed greater ECM protein deposition and accelerated chondrocytic differentiation than MSCs in static cultures [81,82].

Developing engineered bone with vasculature is necessary for graft survival after implantation. Fluid flow-induced shear is a recognized regulator of the vasculature *in vivo*, and many studies have demonstrated favorable effects of shear on vasculogenesis. Vascular endothelial cells under fluid flow remodel stress fibers, change alignment, and upregulate signaling factors involved in vasoregulation [83,84]. Increases in vessel forming gene expression and the formation of vessel-like tubes have been found in co-culture studies, but the influence of fluid flow on these cultures in largely unknown. In one co-culture study, perfusion flow increased uniform endothelial cell distribution and increased the length of endothelial aggregates, which may lead to formation of connected vasculature [85].

## 6. Conclusions

Significant progresses have been made in the field of skeletal regenerative medicine using fluid flows. Studies in 2D flows have shown evidences of flow-induced bone forming cell proliferation and differentiation and regulations over mechanosensitive signaling molecules (ATP, Ca^2+^, PGE2, Cox-2, ERK, *etc.*) and bone specific regulatory molecules (Runx2, AP, BMP-2, OP, OPG, *etc.*). Mechanistic studies using 2D flows have highlighted the role of focal adhesion signaling (FAK) and cytoskeletal tension signaling (RhoA/ROCK) in shear force sensing under flow stimuli. Studies using 3D flows have shown promising data for bone tissue engineering, and demonstrated similar control over various bone specific genes and mineral deposition in the course of flow-driven new bone formation as with 2D flows. Altered flow shear stress conditions and improved transport of soluble factors have been proposed as key factors in 3D flow through porous scaffold, but the contribution of each factor is yet to be fully distinguished. Also, 3D flows for bone tissue engineering still lack knowledge on the effects of scaffold characteristics and co-cultures. While mechanistic data obtained from 2D flows may be utilized to engineer improved 3D bone tissue constructs, it would be better if mechanotransduction pathways are revealed under more physiologically relevant 3D flow conditions. The microfluidics approach is well suited for screening bone cell differentiation capability under varying mechanical and soluble inputs and also for mechanotransduction signaling studies at a high throughput rate. Since microfluidics can provide channels having sizes relevant to microscale *in vivo* interstitial flows, the microfluidics applications may provide unprecedented data for bone forming cell mechanobiology and physiology and therefore for skeletal regenerative medicine. Combined, with proper understanding of the mechanisms of the fluid flow control of bone forming cells and with optimal 3D flow conditions established for improved bone tissue engineering, fluid flow cues will serve as a very powerful tool in advancing skeletal regenerative medicine.

## References

[1] Riddle R.C., Donahue H.J. (2009). From streaming-potentials to shear stress: 25 years of bone cell mechanotransduction. J. Orthop. Res..

[2] Wu H.W., Lin C.C., Lee G.B. (2011). Stem cells in microfluidics. Biomicrofluidics.

[3] Bennett M.R., Hasty J. (2009). Microfluidic devices for measuring gene network dynamics in single cells. Nat. Rev. Genet..

[4] Li J., Rose E., Frances D., Sun Y., You L. (2012). Effect of oscillating fluid flow stimulation on osteocyte mRNA expression. J. Biomech..

[5] Rumney R.M., Sunters A., Reilly G.C., Gartland A. (2012). Application of multiple forms of mechanical loading to human osteoblasts reveals increased ATP release in response to fluid flow in 3D cultures and differential regulation of immediate early genes. J. Biomech..

[6] Lu X.L., Huo B., Chiang V., Guo X.E. (2012). Osteocytic network is more responsive in calcium signaling than osteoblastic network under fluid flow. J. Bone Miner. Res..

[7] Ponik S.M., Triplett J.W., Pavalko F.M. (2007). Osteoblasts and osteocytes respond differently to oscillatory and unidirectional fluid flow profiles. J. Cell. Biochem..

[8] Genetos D.C., Kephart C.J., Zhang Y., Yellowley C.E., Donahue H.J. (2007). Oscillating fluid flow activation of gap junction hemichannels induces ATP release from MLO-Y4 osteocytes. J. Cell. Physiol..

[9] Gardinier J.D., Majumdar S., Duncan R.L., Wang L. (2009). Cyclic hydraulic pressure and fluid flow differentially modulate cytoskeleton re-organization in MC3T3 osteoblasts. Cell. Mol. Bioeng..

[10] Jackson W.M., Jaasma M.J., Tang R.Y., Keaveny T.M. (2008). Mechanical loading by fluid shear is sufficient to alter the cytoskeletal composition of osteoblastic cells. Am. J. Physiol. Cell. Physiol..

[11] Donahue H.J. (2000). Gap junctions and biophysical regulation of bone cell differentiation. Bone.

[12] Hoey D.A., Kelly D.J., Jacobs C.R. (2011). A role for the primary cilium in paracrine signaling between mechanically stimulated osteocytes and mesenchymal stem cells. Biochem. Biophys. Res. Commun..

[13] Taylor A.F., Saunders M.M., Shingle D.L., Cimbala J.M., Zhou Z., Donahue H.J. (2007). Mechanically stimulated osteocytes regulate osteoblastic activity via gap junctions. Am. J. Physiol. Cell. Physiol..

[14] Kulkarni R.N., Bakker A.D., Everts V., Klein-Nulend J. (2010). Inhibition of osteoclastogenesis by mechanically loaded osteocytes: involvement of MEPE. Calcif. Tissue Int..

[15] Kim C.H., You L., Yellowley C.E., Jacobs C.R. (2006). Oscillatory fluid flow-induced shear stress decreases osteoclastogenesis through RANKL and OPG signaling. Bone.

[16] Malone A.M., Batra N.N., Shivaram G., Kwon R.Y., You L., Kim C.H., Rodriguez J., Jair K., Jacobs C.R. (2007). The role of actin cytoskeleton in oscillatory fluid flow-induced signaling in MC3T3-E1 osteoblasts. Am. J. Physiol. Cell. Physiol..

[17] Pavalko F.M., Chen N.X., Turner C.H., Burr D.B., Atkinson S., Hsieh Y.F., Qiu J., Duncan R.L. (1998). Fluid shear-induced mechanical signaling in MC3T3-E1 osteoblasts requires cytoskeleton-integrin interactions. Am. J. Physiol..

[18] Morris H.L., Reed C.I., Haycock J.W., Reilly G.C. (2010). Mechanisms of fluid-flow-induced matrix production in bone tissue engineering. Proc. Inst. Mech. Eng. H.

[19] Kapur S., Baylink D.J., Lau K.H. (2003). Fluid flow shear stress stimulates human osteoblast proliferation and differentiation through multiple interacting and competing signal transduction pathways. Bone.

[20] Li P., Ma Y.C., Sheng X.Y., Dong H.T., Han H., Wang J., Xia Y.Y. (2012). Cyclic fluid shear stress promotes osteoblastic cells proliferation through ERK5 signaling pathway. Mol. Cell. Biochem..

[21] Kreke M.R., Huckle W.R., Goldstein A.S. (2005). Fluid flow stimulates expression of osteopontin and bone sialoprotein by bone marrow stromal cells in a temporally dependent manner. Bone.

[22] Scaglione S., Wendt D., Miggino S., Papadimitropoulos A., Fato M., Quarto R., Martin I. (2008). Effects of fluid flow and calcium phosphate coating on human bone marrow stromal cells cultured in a defined 2D model system. J. Biomed. Mater. Res. A.

[23] Arnsdorf E.J., Tummala P., Kwon R.Y., Jacobs C.R. (2009). Mechanically induced osteogenic differentiation-the role of RhoA, ROCKII and cytoskeletal dynamics. J. Cell. Sci..

[24] Yourek G., McCormick S.M., Mao J.J., Reilly G.C. (2010). Shear stress induces osteogenic differentiation of human mesenchymal stem cells. Regen. Med..

[25] Lim J.Y., Loiselle A.E., Lee J.S., Zhang Y., Salvi J.D., Donahue H.J. (2011). Optimizing the osteogenic potential of adult stem cells for skeletal regeneration. J. Orthop. Res..

[26] Poudel I., Menter D., Lim J.Y. (2012). Directing cell function and fate via micropatterning: Role of cell patterning size, shape, and interconnectivity. Biomed. Eng. Lett..

[27] Salvi J.D., Lim J.Y., Donahue H.J. (2010). Increased mechanosensitivity of cells cultured on nanotopographies. J. Biomech..

[28] Takai E., Landesberg R., Katz R.W., Hung C.T., Guo X.E. (2006). Substrate modulation of osteoblast adhesion strength, focal adhesion kinase activation, and responsiveness to mechanical stimuli. Mol. Cell. Biomech..

[29] Schwartz Z., Denison T.A., Bannister S.R., Cochran D.L., Liu Y.H., Lohmann C.H., Wieland M., Boyan B.D. (2007). Osteoblast response to fluid induced shear depends on substrate microarchitecture and varies with time. J. Biomed. Mater. Res. A.

[30] Damsky C.H., Ilic D. (2002). Integrin signaling: It’s where the action is. Curr. Opin. Cell. Biol..

[31] Lim J.Y., Taylor A.F., Li Z., Vogler E.A., Donahue H.J. (2005). Integrin expression and osteopontin regulation in human fetal osteoblastic cells mediated by substratum surface characteristics. Tissue Eng..

[32] Lim J.Y., Dreiss A.D., Zhou Z., Hansen J.C., Siedlecki C.A., Hengstebeck R.W., Cheng J., Winograd N., Donahue H.J. (2007). The regulation of integrin-mediated osteoblast focal adhesion and focal adhesion kinase expression by nanoscale topography. Biomaterials.

[33] Young S.R., Gerard-O’Riley R., Kim J.B., Pavalko F.M. (2009). Focal adhesion kinase is important for fluid shear stress-induced mechanotransduction in osteoblasts. J. Bone Miner. Res..

[34] Young S.R., Hum J.M., Rodenberg E., Turner C.H., Pavalko F.M. (2011). Non-overlapping functions for Pyk2 and FAK in osteoblasts during fluid shear stress-induced mechanotransduction. PLoS One.

[35] Myers K.A., Rattner J.B., Shrive N.G., Hart D.A. (2007). Osteoblast-like cells and fluid flow: cytoskeleton-dependent shear sensitivity. Biochem. Biophys. Res. Commun..

[36] Sterck J.G., Klein-Nulend J., Lips P., Burger E.H. (1998). Response of normal and osteoporotic human bone cells to mechanical stress *in vitro*. Am. J. Physiol..

[37] Bakker A.D., Klein-Nulend J., Tanck E., Heyligers I.C., Albers G.H., Lips P., Burger E.H. (2006). Different responsiveness to mechanical stress of bone cells from osteoporotic *versus* osteoarthritic donors. Osteoporos. Int..

[38] Zhao F., Chella R., Ma T. (2007). Effects of shear stress on 3-D human mesenchymal stem cell construct development in a perfusion bioreactor system: Experiments and hydrodynamic modeling. Biotechnol. Bioeng..

[39] Barron M.J., Tsai C.J., Donahue S.W. (2010). Mechanical stimulation mediates gene expression in MC3T3 osteoblastic cells differently in 2D and 3D environments. J. Biomech. Eng..

[40] Meinel L., Karageorgiou V., Fajardo R., Snyder B., Shinde-Patil V., Zichner L., Kaplan D., Langer R., Vunjak-Novakovic G. (2004). Bone tissue engineering using human mesenchymal stem cells: effects of scaffold material and medium flow. Ann. Biomed. Eng..

[41] Riehl B.D., Park J.H., Kwon I.K., Lim J.Y. (2012). Mechanical stretching for tissue engineering: Two-dimensional and three-dimensional constructs. Tissue Eng. Part. B Rev..

[42] Bjerre L., Bunger C., Baatrup A., Kassem M., Mygind T. (2011). Flow perfusion culture of human mesenchymal stem cells on coralline hydroxyapatite scaffolds with various pore sizes. J. Biomed. Mater. Res. A.

[43] Sikavitsas V.I., Bancroft G.N., Holtorf H.L., Jansen J.A., Mikos A.G. (2003). Mineralized matrix deposition by marrow stromal osteoblasts in 3D perfusion culture increases with increasing fluid shear forces. Proc. Natl. Acad. Sci. USA.

[44] Kim J., Ma T. (2012). Bioreactor strategy in bone tissue engineering: pre-culture and osteogenic differentiation under two flow configurations. Tissue Eng. Part. A.

[45] Liu L., Yu B., Chen J., Tang Z., Zong C., Shen D., Zheng Q., Tong X., Gao C., Wang J. (2012). Different effects of intermittent and continuous fluid shear stresses on osteogenic differentiation of human mesenchymal stem cells. Biomech. Model. Mechanobiol..

[46] McCoy R.J., Jungreuthmayer C., O'Brien F.J. (2012). Influence of flow rate and scaffold pore size on cell behavior during mechanical stimulation in a flow perfusion bioreactor. Biotechnol. Bioeng..

[47] Jungreuthmayer C., Donahue S.W., Jaasma M.J., Al-Munajjed A.A., Zanghellini J., Kelly D.J., O'Brien F.J. (2009). A comparative study of shear stresses in collagen-glycosaminoglycan and calcium phosphate scaffolds in bone tissue-engineering bioreactors. Tissue Eng. Part. A.

[48] Fritton S.P., Weinbaum S. (2009). Fluid and solute transport in bone: flow-induced mechanotransduction. Annu. Rev. Fluid Mech..

[49] Price C., Zhou X., Li W., Wang L. (2011). Real-time measurement of solute transport within the lacunar-canalicular system of mechanically loaded bone: direct evidence for load-induced fluid flow. J. Bone Miner. Res..

[50] Schmidt S.M., McCready M.J., Ostafin A.E. (2005). Effect of oscillating fluid shear on solute transport in cortical bone. J. Biomech..

[51] Porter B., Zauel R., Stockman H., Guldberg R., Fyhrie D. (2005). 3-D computational modeling of media flow through scaffolds in a perfusion bioreactor. J. Biomech..

[52] Kim J., Ma T. (2012). Perfusion regulation of hMSC microenvironment and osteogenic differentiation in 3D scaffold. Biotechnol. Bioeng..

[53] Li D., Tang T., Lu J., Dai K. (2009). Effects of flow shear stress and mass transport on the construction of a large-scale tissue-engineered bone in a perfusion bioreactor. Tissue Eng. Part. A.

[54] Riddle R.C., Hippe K.R., Donahue H.J. (2008). Chemotransport contributes to the effect of oscillatory fluid flow on human bone marrow stromal cell proliferation. J. Orthop. Res..

[55] Cukierman E., Pankov R., Stevens D.R., Yamada K.M. (2001). Taking cell-matrix adhesions to the third dimension. Science.

[56] Stiehler M., Bunger C., Baatrup A., Lind M., Kassem M., Mygind T. (2009). Effect of dynamic 3-D culture on proliferation, distribution, and osteogenic differentiation of human mesenchymal stem cell. J. Biomed. Mater. Res. A.

[57] Fröhlich M., Grayson W.L., Marolt D., Gimble J.M., Kregar-Velikonja N., Vunjak-Novakovic G. (2010). Bone grafts engineered from human adipose-derived stem cells in perfusion bioreactor culture. Tissue Eng. Part. A.

[58] Grayson W.L., Marolt D., Bhumiratana S., Frohlich M., Guo X.E., Vunjak-Novakovic G. (2011). Optimizing the medium perfusion rate in bone tissue engineering bioreactors. Biotechnol. Bioeng..

[59] Janssen F.W., van Dijkhuizen-Radersma R., Van Oorschot A., Oostra J., de Bruijn J.D., Van Blitterswijk C.A. (2010). Human tissue-engineered bone produced in clinically relevant amounts using a semi-automated perfusion bioreactor system: a preliminary study. J. Tissue Eng. Regen. Med..

[60] Grayson W.L., Frohlich M., Yeager K., Bhumiratana S., Chan M.E., Cannizzaro C., Wan L.Q., Liu X.S., Guo X.E., Vunjak-Novakovic G. (2010). Engineering anatomically shaped human bone grafts. Proc. Natl. Acad. Sci. USA.

[61] Nakamura M., Iwanaga S., Henmi C., Arai K., Nishiyama Y. (2010). Biomatrices and biomaterials for future developments of bioprinting and biofabrication. Biofabrication..

[62] Liao J., Guo X., Nelson D., Kasper F.K., Mikos A.G. (2010). Modulation of osteogenic properties of biodegradable polymer/extracellular matrix scaffolds generated with a flow perfusion bioreactor. Acta. Biomater..

[63] Pham Q.P., Kasper F.K., Mistry A.S., Sharma U., Yasko A.W., Jansen J.A., Mikos A.G. (2009). Analysis of the osteoinductive capacity and angiogenicity of an *in vitro* generated extracellular matrix. J. Biomed. Mater. Res. A.

[64] Wheeler A.R., Throndset W.R., Whelan R.J., Leach A.M., Zare R.N., Liao Y.H., Farrell K., Manger I.D., Daridon A. (2003). microfluidic device for single-cell analysis. Anal. Chem..

[65] Thomas R.S., Mitchell P.D., Oreffo R.O., Morgan H. (2010). Trapping single human osteoblast-like cells from a heterogeneous population using a dielectrophoretic microfluidic device. Biomicrofluidics.

[66] Kou S., Pan L., van Noort D., Meng G., Wu X., Sun H., Xu J., Lee I. (2011). A multishear microfluidic device for quantitative analysis of calcium dynamics in osteoblasts. Biochem. Biophys. Res. Commun..

[67] Leclerc E., David B., Griscom L., Lepioufle B., Fujii T., Layrolle P., Legallaisa C. (2006). Study of osteoblastic cells in a microfluidic environment. Biomaterials.

[68] Jang K., Sato K., Igawa K., Chung U.I., Kitamori T. (2008). Development of an osteoblast-based 3D continuous-perfusion microfluidic system for drug screening. Anal. Bioanal. Chem..

[69] Song S.H., Choi J., Jung H.I. (2010). A microfluidic magnetic bead impact generator for physical stimulation of osteoblast cell. Electrophoresis.

[70] Kirchhof K., Andar A., Yin H.B., Gadegaard N., Riehle M.O., Groth T. (2011). Polyelectrolyte multilayers generated in a microfluidic device with pH gradients direct adhesion and movement of cells. Lab. Chip.

[71] Zhang Y., Gazit Z., Pelled G., Gazit D., Vunjak-Novakovic G. (2011). Patterning osteogenesis by inducible gene expression in microfluidic culture systems. Integr. Biol..

[72] Numthuam S., Ginoza T., Zhu M., Suzuki H., Fukuda J. (2011). Gold-black micropillar electrodes for microfluidic ELISA of bone metabolic markers. Analyst.

[73] Chen J., Zheng Y., Tan Q., Shojaei-Baghini E., Zhang Y.L., Li J., Prasad P., You L., Wu X.Y., Sun Y. (2011). Classification of cell types using a microfluidic device for mechanical and electrical measurement on single cells. Lab. Chip.

[74] Lee J.H., Gu Y., Wang H., Lee W.Y. (2012). Microfluidic 3D bone tissue model for high-throughput evaluation of wound-healing and infection-preventing biomaterials. Biomaterials.

[75] Lee J.H., Wang H., Kaplan J.B., Lee W.Y. (2010). Effects of Staphylococcus epidermidis on osteoblast cell adhesion and viability on a Ti alloy surface in a microfluidic co-culture environment. Acta. Biomater..

[76] Wei C.W., Cheng J.Y., Young T.H. (2006). Elucidating *in vitro* cell-cell interaction using a microfluidic coculture system. Biomed. Microdevices..

[77] Scherberich A., Galli R., Jaquiery C., Farhadi J., Martin I. (2007). Three-dimensional perfusion culture of human adipose tissue-derived endothelial and osteoblastic progenitors generates osteogenic constructs with intrinsic vascularization capacity. Stem Cells.

[78] Yuan L., Sakamoto N., Song G., Sato M. (2012). Migration of human mesenchymal stem cells under low shear stress mediated by mitogen-activated protein kinase signaling. Stem Cells Dev..

[79] Gurkan U.A., Akkus O. (2008). The mechanical environment of bone marrow: A review. Ann. Biomed. Eng..

[80] Kshitiz, Park J., Kim P., Helen W., Engler A.J., Levchenko A., Kim D.H. (2012). Control of stem cell fate and function by engineering physical microenvironments. Integr. Biol..

[81] Gonçalves A., Costa P., Rodrigues M.T., Dias I.R., Reis R.L., Gomes M.E. (2011). Effect of flow perfusion conditions in the chondrogenic differentiation of bone marrow stromal cells cultured onto starch based biodegradable scaffolds. Acta. Biomater..

[82] Alves da Silva M.L., Martins A., Costa-Pinto A.R., Correlo V.M., Sol P., Bhattacharya M., Faria S., Reis R.L., Neves N.M. (2011). Chondrogenic differentiation of human bone marrow mesenchymal stem cells in chitosan-based scaffolds using a flow-perfusion bioreactor. J. Tissue Eng. Regen. Med..

[83] Kinney M.A., Sargent C.Y., McDevitt T.C. (2011). The multiparametric effects of hydrodynamic environments on stem cell culture. Tissue Eng. Part. B Rev..

[84] Patwari P., Lee R.T. (2008). Mechanical control of tissue morphogenesis. Circ. Res..

[85] Barron M.J., Goldman J., Tsai C.J., Donahue S.W. (2012). Perfusion flow enhances osteogenic gene expression and the infiltration of osteoblasts and endothelial cells into three-dimensional calcium phosphate scaffolds. Int. J. Biomater..

